# Potential Utility of a 4th-Generation EGFR-TKI and Exploration of Resistance Mechanisms—An In Vitro Study

**DOI:** 10.3390/biomedicines12071412

**Published:** 2024-06-25

**Authors:** Shota Fukuda, Kenichi Suda, Akira Hamada, Hana Oiki, Shuta Ohara, Masaoki Ito, Junichi Soh, Tetsuya Mitsudomi, Yasuhiro Tsutani

**Affiliations:** Division of Thoracic Surgery, Department of Surgery, Kindai University Faculty of Medicine, Osaka-Sayama 589-8511, Japan; shota-fukuda@med.kindai.ac.jp (S.F.); a-hamada@med.kindai.ac.jp (A.H.); hana.oiki@med.kindai.ac.jp (H.O.); 154148@med.kindai.ac.jp (S.O.); masito@med.kindai.ac.jp (M.I.); drjsou7@gmail.com (J.S.); mitsudom@gmail.com (T.M.); tsutani@med.kindai.ac.jp (Y.T.)

**Keywords:** *EGFR* mutation, osimertinib, acquired resistance, secondary mutations, novel drugs, non-small-cell lung cancer, epithelial-to-mesenchymal transition, *MET* gene amplification, Ba/F3 cells

## Abstract

The emergence of acquired resistance to EGFR-tyrosine kinase inhibitors (TKIs) is almost inevitable even after a remarkable clinical response. Secondary mutations such as T790M and C797S are responsible for the resistance to 1st/2nd-generation (1/2G) TKIs and 3G TKIs, respectively. To overcome both the T790M and C797S mutations, novel 4G EGFR-TKIs are now under early clinical development. In this study, we evaluated the efficacy of a 4G EGFR-TKI in the treatment of lung cancer with *EGFR* mutation as well as explored resistance mechanisms to a 4G TKI. First, we compared the efficacies of seven TKIs including a 4G TKI, BI4020, against Ba/F3 cell models that simulate resistant tumors after front-line osimertinib treatment failure because of a secondary mutation. We also established acquired resistant cells to BI4020 by chronic drug exposure. Ba/F3 cells with an osimertinib-resistant secondary mutation were refractory to all 3G TKIs tested (alflutinib, lazertinib, rezivertinib, almonertinib, and befotertinib). BI4020 inhibited the growth of C797S-positive cells; however, it was not effective against L718Q-positive cells. Erlotinib was active against all Ba/F3 cells tested. In the analysis of resistance mechanisms of BI4020-resistant (BIR) cells, none harbored secondary *EGFR* mutations. HCC827BIR cells had *MET* gene amplification and were sensitive to a combination of capmatinib (MET-TKI) and BI4020. HCC4006BIR and H1975BIR cells exhibited epithelial-to-mesenchymal transition. This study suggests that erlotinib may be more suitable than 4G TKIs to overcome secondary mutations after front-line osimertinib. We found that off-target mechanisms that cause resistance to earlier-generation TKIs will also cause resistance to 4G TKIs.

## 1. Introduction

Epidermal growth factor receptor (EGFR) tyrosine kinase inhibitors (TKIs) are the key drugs in treatment of non-small-cell lung cancer (NSCLC) patients with *EGFR* mutation [1]. Currently, 1st/2nd-generation (1/2G) EGFR-TKIs and a 3rd-generation (3G) TKI, osimertinib [2,3], are available in daily clinical practice. Additionally, several novel 3G EGFR-TKIs are now in clinical development [4,5,6]. However, secondary mutations such as T790M and C797S result in the acquired resistance to 1/2G EGFR-TKIs and 3G EGFR-TKIs, respectively [7,8,9,10]. Therefore, a novel type of EGFR-TKIs, the 4th-generation (4G) EGFR-TKIs, that can overcome both the T790M and C797S mutations, even if they are present in cis, are now under early clinical development [11,12].

**Figure 1:**
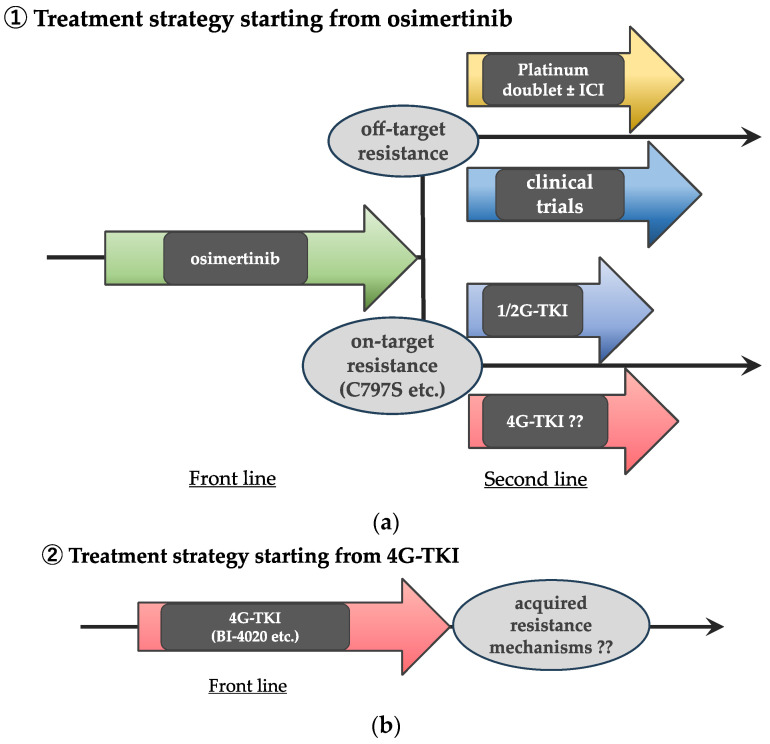
**Potential future treatment strategies using 4G EGFR-TKIs.** (**a**) Treatment strategies starting from osimertinib, with 4G-TKI as a second-line. In this scenario, TKIs that can overcome on-target osimertinib resistance should be used as the second-line TKI. In case of off-target resistance, platinum doublet with/without immune checkpoint inhibitor (ICI) or enrollment to clinical trials would be the options since EGFR-TKI monotherapy will no longer be active. (**b**) 4G-TKI as a front-line treatment. If 4G EGFR-TKIs will be used as a front-line setting, exploration of its resistance mechanisms is needed.

Many of the current guidelines recommend the 3G TKI osimertinib as a front-line treatment for *EGFR*-mutated NSCLC patients, as a result of the significant improvement in prolonged progression-free and overall survival with osimertinib compared with 1G EGFR-TKIs in the FLAURA Phase III trial [2,13]. Therefore, treatment strategies involving 4G EGFR-TKIs would be either (a) as a second-line treatment after front-line osimertinib failure or (b) as a front-line therapy for *EGFR*-mutated NSCLC (Figure 1). In the case of the former treatment strategy, the optimal second-line treatment depends on the type(s) of acquired resistance mechanism(s). However, in daily clinical practice, it is sometimes difficult to perform re-biopsy at the time of acquisition of resistance [14]; furthermore, there may be heterogeneity in molecular mechanisms of acquired resistance [15,16]. Therefore, as a second-line TKI, drug(s) that can overcome a wider range of possible secondary mutations are desirable.

In this study, we compared the efficacy of a 4G EGFR-TKI with a 1G EGFR-TKI (erlotinib) and several 3G EGFR-TKIs against in vitro cell models harboring secondary mutations that may cause acquired resistance to front-line osimertinib. We also explored acquired resistance mechanisms to the 4G EGFR-TKI using in vitro cell models with acquired resistance to the 4G EGFR-TKI after chronic drug exposure.

## 2. Materials and Methods

### 2.1. Exploration of Acquired Resistance Mechanisms to Front-Line Osimertinib

**Figure 2:**
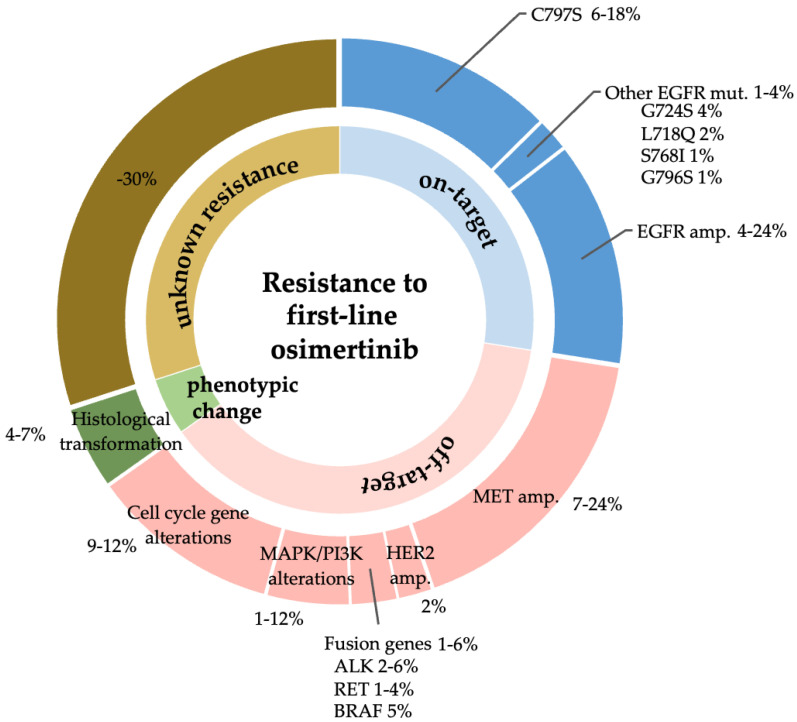
**Summary of acquired resistance mechanisms to front-line osimertinib.** Data are summarized from publications that evaluated acquired resistance mechanisms to front-line osimertinib [10,18,19,20,21,22,23,24,25,26,27].

Acquired resistance mechanisms to EGFR-TKIs are classified as on-target mechanisms (mainly from secondary *EGFR* mutations), off-target mechanisms (activation of a bypass signaling pathway), and histological transformation [17]. Acquired resistance mechanisms to front-line osimertinib were searched using PubMed in December 2022. Acquired resistance mutations that were reported only at second-line osimertinib failure were excluded. In the seven publications that met the inclusion criteria [18,19,20,21,22,23,24], we identified four secondary mutations (C797S, G724S, L718Q, and S768I) as secondary mutations that may cause acquired resistance to front-line osimertinib. Data of acquired resistance mechanisms to front-line osimertinib were updated at the time of writing by adding four publications that were reported after December 2022 [10,25,26,27] (Figure 2).

### 2.2. Cell Lines and Reagents

Because there are no commercially available NSCLC cell lines harboring an *EGFR*-activating mutation plus one of secondary mutations, we used Ba/F3 cells as the model with oncogene dependence and vulnerability to specific targeted therapies (i.e., cells whose proliferation and survival depend on mutated EGFR). Ba/F3 cell lines harboring one of C797S, G724S, or L718Q secondary mutations were established in our previous study [28]. In this study, we planned to establish Ba/F3 cells harboring S768I as the secondary mutation as previously described [28,29]. 

*EGFR*-mutated lung cancer cell lines (HCC827, HCC4006, PC9, and H1975) were cultured in RPMI 1640 medium (Wako, Osaka, Japan) with 10% fetal bovine serum (Sigma-Aldrich, St. Louis, MO, USA). Cell authentication was confirmed using STR profiling as described in our previous studies [30,31,32]. BI4020-resistant lung cancer cells were established by chronic exposure to BI4020 at increasing concentrations from 1 nM to 2000 nM as previously described [30,31].

We purchased the following compounds from Selleck Chemicals (Houston, TX, USA): 1G EGFR-TKI (erlotinib; S7786), 3G EGFR-TKIs (osimertinib; S7297, alflutinib; S6868, lazertinib; S8724, almonertinib; S8817), and 4G EGFR-TKI (BI4020; S8921). The 3G EGFR-TKIs rezivertinib (HY-109189) and befotertinib (HY-137433) were purchased from MedChemExpress (Monmouth Junction, NJ, USA). Each compound was dissolved in DMSO (Sigma-Aldrich, St. Louis, MO, USA).

### 2.3. Growth Inhibition Assay

Growth inhibition assay was performed as described previously [29,30,33]. Briefly, Ba/F3 cells or lung cancer cells were seeded at a density of 2–3 × 10^3^/well in 96-well plates in RPMI 1640 medium supplemented with 10% FBS and cultured for 24 h. Then, DMSO or a designated drug(s) at the indicated concentration was added. Medium-only wells without seeded cells were also prepared and used as a blank. After 72 h, the Cell Counting Kit-8 reagent (Dojindo Laboratories, Kumamoto, Japan) was added in each well, and the plates were incubated for 2–4 h. The absorbance at 450 nm, which is correlated with the numbers of alive cells, was read using a multiplate reader (Tecan, Mannedorf, Switzerland) following the manufacturer’s protocol, and then, the IC_50_ values were calculated. The efficacy of each drug was also assessed using the sensitivity index (SI) as previously described [29]. The SI was defined as the IC_50_ value divided by the trough concentration of a given drug (IC_50_/Ctrough max 100) at the recommended dose in clinical trials.

### 2.4. Western Blot Analysis

Cells were treated with indicated concentrations of drug for 24 h. The cells were then washed twice with phosphate-buffered saline and lysed in lysis buffer. Protein content was determined using a bicinchoninic acid protein assay (Bio-Rad, Hercules, CA, USA); samples were electrophoresed and transferred to polyvinylidene difluoride membranes (Bio-Rad). Immunoblotting was performed using the Transblot Turbo Transfer System (Bio-Rad) following the manufacturer’s instructions. The membranes were probed using one of following antibodies overnight at 4 °C: phosphor-MET (#3126), total-MET (#8198), p-Erk (#4370), E-cadherin (#3195), Vimentin (#5741), and β-actin (#4970), which were purchased from Cell Signaling Technology (Danvers, MA, USA). Blocking buffer and antibody solutions were obtained from Takara (Kusatsu, Japan). Horseradish peroxidase-conjugated secondary anti-rabbit immunoglobulin G (#7074, Cell Signaling Technology) was used as the secondary antibody. In the chemiluminescence assay, the membranes were reacted with enhanced chemiluminescence solution (GE Healthcare, Chicago, IL, USA) and scanned by an Amersham imager 680 (GE Healthcare) as previously described [29].

### 2.5. Human Phospho-RTK Array

The relative phosphorylation levels of 43 receptor-tyrosine kinases (RTK) were screened using the Human Phospho-RTK Array Kit (R&D Systems, Minneapolis, MN, USA) as previously described [30]. Briefly, PC9 parental and resistant cells were cultured in RPMI-1640 containing 10% FBS and 4000 nM of BI4020 for 72 h. Cells were lysed in array buffer prior to reaching confluence. The arrays were blocked with blocking buffer and incubated with 200 µg cell lysate overnight at 4 °C. The arrays were washed and incubated with a horseradish peroxidase (HRP)-conjugated phospho-kinase antibody, and the chemiluminescence was detected.

### 2.6. Mutation Analysis and Gene Copy Number Analysis

Total RNA was prepared using an RNeasy^®^ Plus Mini kit (250) (Qiagen, Hilden, Germany), following the manufacturer’s protocol. Random-primed, first-strand cDNA was synthesized from 10 µg of total RNA using the Rever Tra Ace^®^ qPCR·RT kit (TOYOBO, Osaka, Japan), following the manufacturer’s instructions. Mutation analysis of exons 18 to 21 of the *EGFR* gene was carried out by direct sequencing using primer sets as previously described [30].

Genomic DNA was extracted using a DNeasy^®^ Blood & Tissue Kit (250) (Qiagen), following the manufacturer’s protocol. The copy number of the *MET* gene relative to a *LINE-1* repetitive element was measured by quantitative real-time PCR using the SYBR Green Method (Power SYBR Green PCR Master Mix; Qiagen) as described previously [30]. PCR was performed in triplicate for each primer set. Normal genomic DNA was used as a standard sample.

## 3. Results

### 3.1. Efficacy of BI4020 and Other TKIs against Osimertinib-Resistant Secondary Mutations 

**Figure 3:**
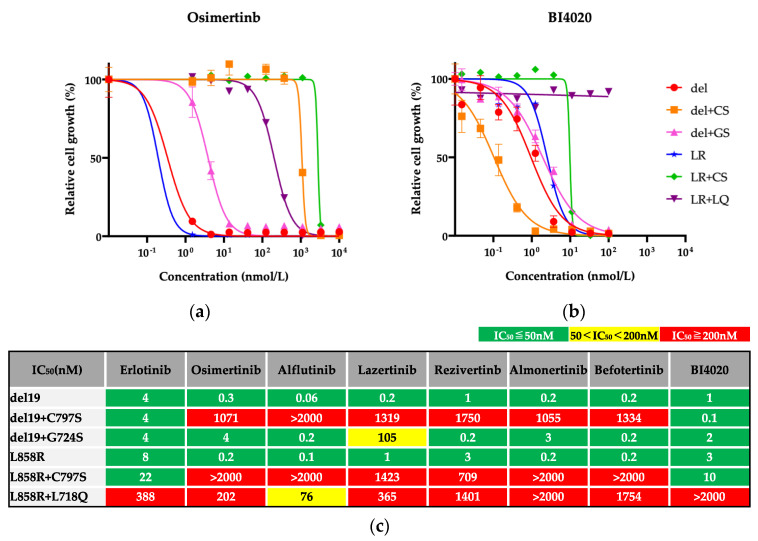

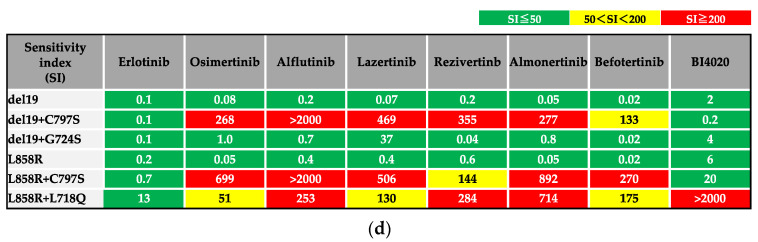
**Growth inhibitory assay of various EGFR TKIs against Ba/F3 cells harboring one of the osimertinib resistance secondary mutations.** Growth inhibitory curves of osimertinib (**a**) and BI4020 (**b**) in all cells tested. IC_50_ values (**c**) and sensitivity index (**d**) of each TKI were summarized.

Because we could not establish IL-3-independent Ba/F3 cells with S768I secondary mutation, we evaluated drug efficacies using the other Ba/F3 cells. The efficacy of a 1G EGFR-TKI (erlotinib), novel 3G EGFR-TKIs (alflutinib, lazertinib, rezivertinib, almonertinib, befotertinib), and a 4G EGFR-TKI (BI4020) against Ba/F3 cells harboring one of the osimertinib-resistant secondary mutations, as well as those with activating mutation alone, was evaluated. The secondary mutations conferred insensitivity to osimertinib (Figure 3a). In contrast, many of the parental and resistant cell lines responded similarly to BI4020 (similar IC_50_ values and SI), except for Ba/F3 cells with L858R+L718Q mutations (Figure 3b). The IC_50_ values of each drug are summarized in Figure 3c.

In the evaluation of SI (Figure 3d), we found that all 3G TKIs could not overcome the C797S secondary mutation. Additionally, Ba/F3 cells with the L718Q secondary mutation were insensitive to all 3G TKIs and BI4020. In contrast, erlotinib was effective in all Ba/F3 cells tested. These results suggest that erlotinib may be the most useful TKI after front-line osimertinib failure from on-target resistance mechanisms.

### 3.2. Exploration of Acquired Resistance Mechanisms to Front-Line BI4020

**Figure 4:**
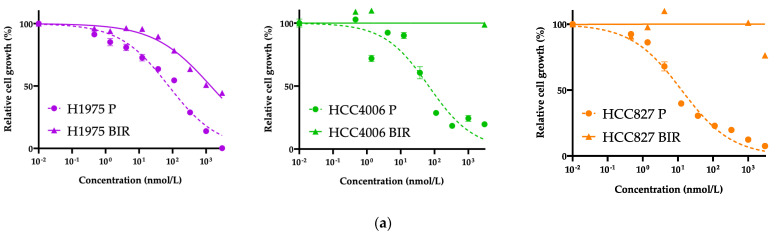

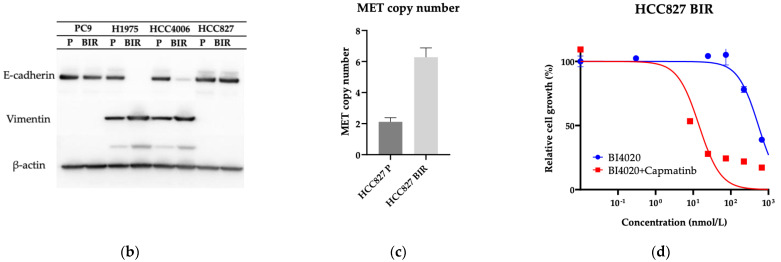
**Establishment of acquired BI4020-resistant cell lines from *EGFR*-mutated TKI-sensitive lung cancer cell lines.** (**a**) Growth inhibitory assay for BI4020 in lung cancer cell lines (H1975, HCC4006, and HCC827) that acquired resistance to BI4020 (BIR) and the parental cells (P). (**b**) Western blotting revealed that H1975 BIR and HCC4006 BIR acquired EMT features. (**c**) Real-time PCR revealed *MET* gene amplification in HCC827 BIR cells (HCC827 parental cells: 2.11 copies, HCC827 BIR: 6.28 copies). (**d**) Capmatinib, a MET-TKI, restored sensitivity to BI4020 in HCC827BIR cells.

**Figure 5:**
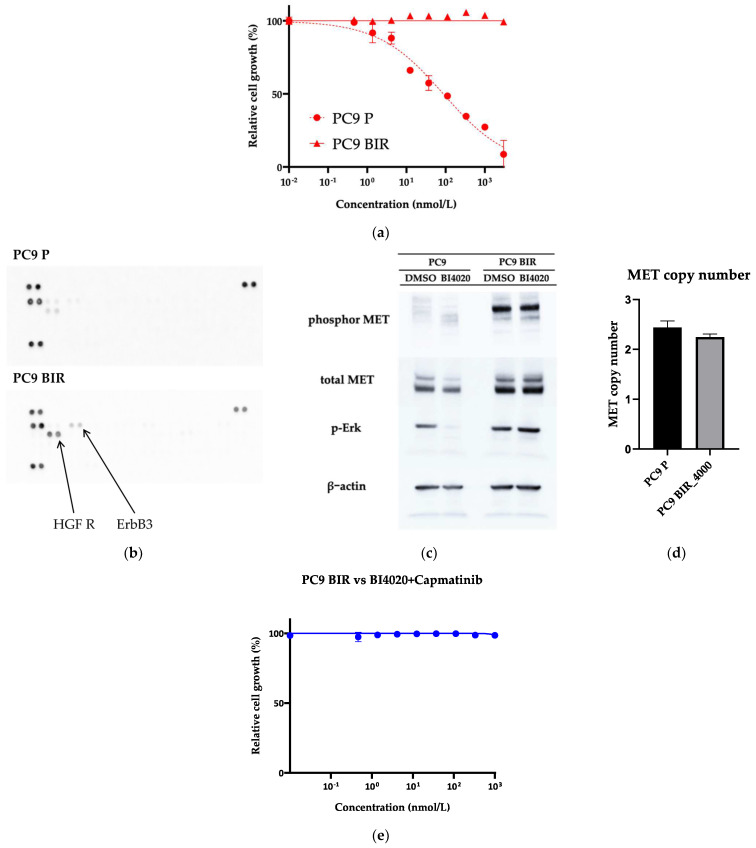
**Establishment of acquired BI4020-resistant cell line from the PC9 lung cancer cell line.** (**a**) Growth inhibitory assay of PC9 parental cells (P) and PC9 BIR cells against the indicated concentrations of BI4020. (**b**) Human phosphor-RTK array in PC9 P and PC9 BIR cells treated with 4000 nM of BI4020 for 72 h. (**c**) Western blotting of PC9 and PC9 BIR cells showed increased total MET and phosphor-MET expression in PC9 BIR cells. (**d**) Real-time PCR showed that PC9 BIR did not acquire *MET* gene copy number gain. (**e**) Growth inhibitory assay of PC9 BIR cells treated with the combination of a MET-TKI (capmatinib) plus BI4020.

We successfully established acquired BI4020-resistant (BIR) cells from PC9, H1975, HCC4006, and HCC827 lung cancer cell lines by chronic exposure of cells to BI4020 at increasing concentrations (Figure 4a and Figure 5a). We first examined any potential secondary mutations of the *EGFR* kinase domain (exons 18–21) in the four resistant lines; no secondary mutation was detected. We and other groups have reported that HCC4006 and H1975 cells often acquire TKI resistance via epithelial–mesenchymal transition (EMT) [17,31,32,34,35,36,37,38]. Because the morphological changes of these resistant cells were similar to those previously reported, we further evaluated E-cadherin and vimentin expression. As shown in Figure 4b, both H1975BIR and HCC4006BIR cells, but not HCC827BIR cells, exhibited EMT.

On the other hand, *MET* gene amplification has been reported as an acquired resistant mechanism to EGFR-TKIs in HCC827 cells [17,30,39,40,41,42]. Therefore, we examined *MET* gene copy number in HCC827BIR cells and HCC827 parental cells using real-time PCR. As shown in Figure 4c, *MET* gene copy number was increased in HCC827BIR cells. The combination treatment with capmatinib, a MET-TKI, plus BI4020 was effective in inhibiting the growth of HCC827BIR cells compared with BI4020 alone (Figure 4d).

Various resistance mechanisms to 1–3G TKIs have been reported in PC9 cells. PC9 BIR cells did not harbor a secondary *EGFR* mutation, and thus, we used a human phosphor-RTK assay. As shown in Figure 5b, HGF-R (MET) phosphorylation was increased in PC9 BIR cells compared with PC9 parental cells. This result was confirmed by Western blot (Figure 5c). However, we did not observe *MET* gene copy number gain in PC9 BIR cells; furthermore, either the type Ib MET-TKI capmatinib (Figure 5e) or the type II MET-TKI foretinib did not restore BI4020 sensitivity in PC9 BIR cells. This result suggests that increased MET protein expression (either total or phosphorylated MET) does not necessarily mean that MET activation is the mechanism of acquired resistance to EGFR-TKIs.

## 4. Discussion

The T790M mutation in the *EGFR* gene is the most frequent mechanism of acquired resistance to 1G or 2G EGFR-TKIs, present in ~50% of all NSCLC cases. Initially, 3G EGFR-TKIs that can overcome T790M-mediated resistance have been developed for use in a second-line setting after treatment failure of 1G EGFR-TKIs [43,44]. Several studies have reported that a tertiary C797S mutation (mainly in cis), which impairs the covalent binding between the cysteine residue at position 797 of EGFR and 3G EGFR-TKI, induces acquisition of resistance to second-line 3G EGFR-TKIs [18,45,46]. Therefore, to inhibit the growth of *EGFR*-mutated NSCLC cells with triple *EGFR* mutations, 4G EGFR-TKIs have been developed [11,12]. In addition to BI-4020 that was used in our study, various 4G EGFR-TKIs are now being studied. Yun et al. reported that JIN-02 inhibited cell growth to a greater degree than osimertinib in an EGFR 19Del/T790M/C797S in cis model (IC_50_ values: 92.1 nM vs. > 4000 nM) [47]. Sun et al. reported that the IC_50_ values of BBT-176 against EGFR 19Del/C797S, EGFR 19Del/T790M/C797S, and EGFR L858R/C797S were 4.36, 1.79, and 5.35 nmol/L, respectively, while the values were 304.39, 124.82, and 573.72 nmol/L for osimertinib [12]. Based on these promising in vitro data, several clinical trials using 4G EGFR-TKI are ongoing, such as the SYMPHONY phase 1/2 trial (NCT04862780) of a 4G EGFR-TKI, BLU-945, as monotherapy or combination therapy of BLU-945 plus osimertinib. This study enrolled *EGFR*-mutated NSCLC patients who had received ≥1 EGFR TKI(s), and 48% of patients experienced tumor regression at doses of 400 mg/day or higher with BLU-945 monotherapy [48].

The great success of osimertinib, a 3G EGFR-TKI, in the FLAURA study [10] led to the recommendation of the use of osimertinib in a front-line setting in many countries. Several recent studies that explored the efficacy and safety of novel front-line combination treatments, such as the FLAURA2 study and MARIPOSA study, also involve a 3G EGFR-TKI as one of the combined agents [49,50,51]. Therefore, there may not be many chances to use 4G EGFR-TKIs as originally expected (i.e., after treatment failures of 1G/2G TKI, and then 3G TKI).

In this study, we evaluated the potential utility of a 4G EGFR-TKI for the treatment of NSCLC with common *EGFR* mutations. However, in our in vitro model reflecting the use of 4G EGFR-TKIs after treatment failure of front-line osimertinib, we observed that erlotinib, a 1G EGFR-TKI, showed wider efficacy than the 4G EGFR-TKI (or other novel 3G EGFR-TKIs) against secondary mutations that emerge after front-line osimertinib treatment. We did not include 2G EGFR-TKIs, such as afatinib or dacomitinib, in this study, because these drugs cannot overcome the most frequent secondary mutation after osimertinib treatment failure, C797S [28]. These results suggest that 1G EGFR-TKI would be a suitable EGFR-TKI following osimertinib treatment failure as previously reported [52,53,54]. However, in clinical practice, cytotoxic agents with/without an immune checkpoint inhibitor are used as second-line treatment after front-line osimertinib treatment failure, because only 25% of patients have on-target resistance mechanisms (Figure 2). Because osimertinib re-challenge is sometimes effective after cytotoxic chemotherapies [55], the use of osimertinib or 1G EGFR-TKI as a re-challenging EGFR-TKI should be discussed [56].

We also explored acquired resistance mechanisms to front-line 4G EGFR-TKI exposure using lung cancer cell lines with activating *EGFR* mutation. While we did not find any secondary mutations in the four established resistant cell lines, we observed *MET* gene amplification (HCC827) and EMT phenotypic change (HCC4006 and H1975) as the mechanisms of resistance to a 4G EGFR-TKI. Because 4G EGFR-TKIs are active against two major secondary mutations, T790M and C797S, it is reasonable that each lung cancer cell line acquired a “preferred” off-target resistance mechanism [17]. Therefore, the results of this study may suggest that, if 4G EGFR-TKIs are approved in clinical practice as front-line therapy, the frequency of on-target acquired resistance mechanisms will further decrease. Moreover, we observed increased phosphorylation of MET in PC9 BIR cells; however, the resistant cells did not acquire *MET* gene copy number gain and, more importantly, the combination of a MET-TKI plus BI4020 did not show efficacy in this resistant cell line. Although we cannot explain why the increased MET phosphorylation was not associated with BI4020 resistance, this result may suggest that gene copy number, but not increased phosphor-MET (or total MET) expression, would be a useful biomarker indicating MET-mediated acquired resistance to EGFR-TKI. This observation would be consistent with the fact that in clinical trials enrolling patients who had developed resistance to EGFR-TKI due to MET aberration(s), some MET immunohistochemistry-positive patients without *MET* gene amplification did not respond to combined EGFR-TKI plus MET-TKI treatment [57,58].

## 5. Conclusions

Our results suggest that erlotinib, but not a 4G EGFR-TKI, may be the most suitable second-line TKI for NSCLC after acquisition of resistance to front-line osimertinib, based on its broad activity against secondary *EGFR* mutations that may emerge after osimertinib treatment failure. Additionally, we observed that lung cancer cells acquire resistance to 4G EGFR-TKI using their “favorite” off-target resistance mechanisms after acquisition of resistance to 1G–3G EGFR-TKIs.

## Data Availability

All data has been published in this manuscript, and further information, if necessary, will be available from the corresponding author upon reasonable requests.
